# Cryptococcosis Associated With Biologic Therapy: A Narrative Review

**DOI:** 10.1093/ofid/ofae316

**Published:** 2024-06-26

**Authors:** Xin Li, Olivier Paccoud, Koon-Ho Chan, Kwok-Yung Yuen, Romain Manchon, Fanny Lanternier, Monica A Slavin, Frank L van de Veerdonk, Tihana Bicanic, Olivier Lortholary

**Affiliations:** Department of Infectious Diseases and Tropical Medicine, Université Paris Cité, Necker-Enfants Malades University Hospital, Assistance Publique–Hôpitaux de Paris, IHU Imagine, Paris, France; Department of Microbiology, School of Clinical Medicine, Li Ka Shing Faculty of Medicine, The University of Hong Kong, Pokfulam, Hong Kong SAR, China; Department of Infectious Diseases and Tropical Medicine, Université Paris Cité, Necker-Enfants Malades University Hospital, Assistance Publique–Hôpitaux de Paris, IHU Imagine, Paris, France; Department of Medicine, School of Clinical Medicine, Li Ka Shing Faculty of Medicine, The University of Hong Kong, Pokfulam, Hong Kong SAR, China; Department of Microbiology, School of Clinical Medicine, Li Ka Shing Faculty of Medicine, The University of Hong Kong, Pokfulam, Hong Kong SAR, China; Department of Infectious Diseases and Tropical Medicine, Université Paris Cité, Necker-Enfants Malades University Hospital, Assistance Publique–Hôpitaux de Paris, IHU Imagine, Paris, France; Department of Infectious Diseases and Tropical Medicine, Université Paris Cité, Necker-Enfants Malades University Hospital, Assistance Publique–Hôpitaux de Paris, IHU Imagine, Paris, France; Institut Pasteur, National Reference Center for Invasive Mycoses and Antifungals, Mycology Translational Research Group, Mycology Department, Université Paris Cité, Paris, France; Department of Infectious Diseases, Peter MacCallum Cancer Centre, Melbourne, Australia; Sir Peter MacCallum Department of Oncology, University of Melbourne, Melbourne, Australia; Victorian Infectious Diseases Service, Royal Melbourne Hospital, Melbourne, Australia; Department of Internal Medicine, Radboud Center for Infectious Diseases, Radboudumc, Nijmegen, the Netherlands; Institute of Infection and Immunity, St George's University of London, London, UK; Department of Infectious Diseases and Tropical Medicine, Université Paris Cité, Necker-Enfants Malades University Hospital, Assistance Publique–Hôpitaux de Paris, IHU Imagine, Paris, France; Institut Pasteur, National Reference Center for Invasive Mycoses and Antifungals, Mycology Translational Research Group, Mycology Department, Université Paris Cité, Paris, France

**Keywords:** autoimmune diseases, biologics, cryptococcosis, hematology, transplant

## Abstract

*Cryptococcus* is an opportunistic fungal pathogen that can cause disseminated infection with predominant central nervous system involvement in patients with compromised immunity. Biologics are increasingly used in the treatment of neoplasms and autoimmune/inflammatory conditions and the prevention of transplant rejection, which may affect human defense mechanisms against cryptococcosis. In this review, we comprehensively investigate the association between cryptococcosis and various biologics, highlighting their risks of infection, clinical manifestations, and clinical outcomes. Clinicians should remain vigilant for the risk of cryptococcosis in patients receiving biologics that affect the Th1/macrophage activation pathways, such as tumor necrosis factor α antagonists, Bruton tyrosine kinase inhibitors, fingolimod, JAK/STAT inhibitors (Janus kinase/signal transducer and activator of transcription), and monoclonal antibody against CD52. Other risk factors—such as age, underlying condition, and concurrent immunosuppressants, especially corticosteroids—should also be taken into account during risk stratification.

## Teaser

In this review, we investigated the association between cryptococcosis and various biologics affecting Th1/macrophage activation pathways, such as tumour necrosis factor-a antagonists, Bruton tyrosine kinase inhibitors, fingolimod, JAK/STAT inhibitors, and monoclonal antibody against CD52, highlighting risks of infection, clinical manifestations, and outcomes

Members of the *Cryptococcus neoformans/gattii* species complex are basidiomycetous fungal pathogens that are environmental saprophytes and the etiologic agents of the potentially fatal human fungal infection cryptococcosis. Clinical manifestation ranges from asymptomatic pulmonary infection to disseminated central nervous system (CNS) infection [1]. Cryptococcosis has become a major global health concern since the HIV pandemic in the 1980s, with most cases occurring in adults infected with HIV who live in sub-Saharan Africa. A recent modeling study estimated 152 000 cases of cryptococcal meningitis occurring among people with HIV per annum, resulting in 112 000 cryptococcosis-related deaths [2]. Besides advanced HIV, other risk factors include hematopoietic stem cell or solid organ transplantation, hematologic malignancies, organ failure, sarcoidosis, primary immunodeficiencies affecting T-cell immunity, autoantibody against the granulocyte-macrophage colony-stimulating factor (GM-CSF), and iatrogenic immunosuppression (eg, corticosteroids) [3].

With advances in the medical treatment of cancer and autoimmune and inflammatory diseases, including wider availability of solid organ and hematopoietic stem cell transplantation and an expanding variety of immunomodulatory agents, the number of patients who are immunocompromised and at risk of opportunistic infections is increasing. In addition, recent modeling studies have demonstrated global warming as a major driver of the expansion in the ecologic niches of pathogenic cryptococci [4]. Coupled with the changing patterns of human behaviors and increasing numbers of susceptible hosts, the incidence of cryptococcosis is expected to rise in the next decades. In this review, we describe the pathobiology of cryptococcosis and review the risks of infection conferred by different biological agents used in clinical practice.

## PATHOBIOLOGY

### Pathogenesis

**Figure 1.**
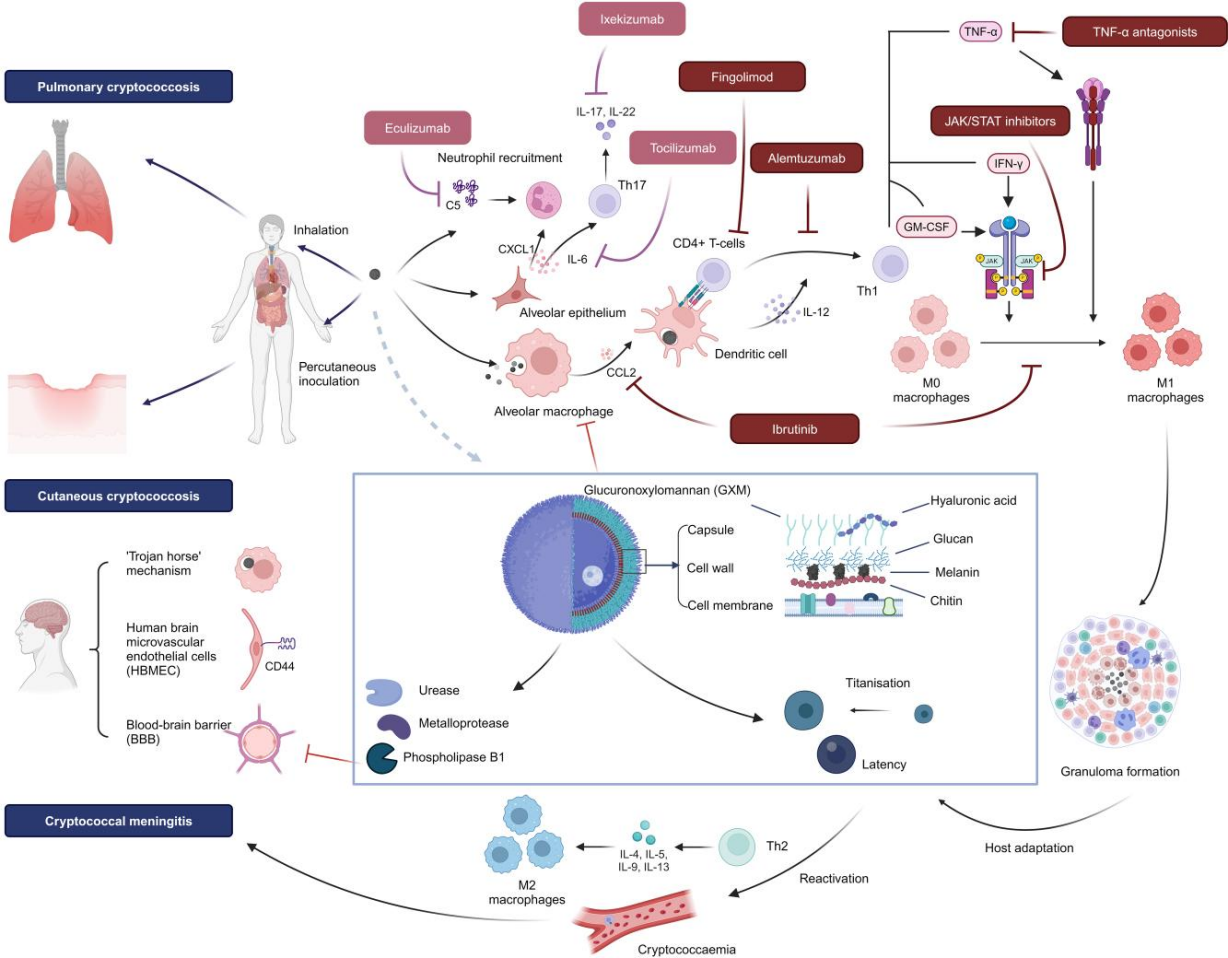
Cryptococcosis pathogenesis and the impact of major categories of biologics in this review [1, 6, 7]. Pathogenetic cryptococci elaborate various virulence factors to help establish infection and dissemination, especially to the central nervous system. For a detailed description of the impact of specific biologics on cryptococcosis, refer to the corresponding sections on TNF-α blockers, Bruton tyrosine kinase inhibitors, fingolimod, and others. CXCL1, chemokine (C-X-C motif) ligand 1; GM-CSF, granulocyte-macrophage colony-stimulating factor; IFN-γ, interferon γ; IL, interleukin; JAK/STAT, Janus kinase/signal transducer and activator of transcription; TNF-α, tumour necrosis factor-α. Image created with BioRender.com.

Cryptococcal infection occurs via inhalation of small desiccated yeast cells or basidiospores (1–5 µm), which reach the lower bronchioalveolar tree [1, 5, 6] (Figure 1). Due to the ubiquitous environmental distribution of *Cryptococcus*, most infections are acquired in early childhood [8]. Primary pulmonary cryptococcosis usually results in asymptomatic or subclinical infection in individuals who are immunocompetent [8, 9] but can result in pneumonia in patients who are immunocompromised [10]. In immunocompetent hosts, cryptococci are either cleared by the immune system after initial infection or establish a latent stage in immune cells, primarily macrophages, that can reactivate later in life due to immune dysregulation [11, 12].

Besides pulmonary infection, disseminated infection involving the skin, soft tissue, bone, joint, liver, lymph nodes, peritoneum, urogenital tract, adrenal, eyes, and especially the CNS can occur, particularly in immunosuppressed hosts [3]. Cryptococcal entry into the CNS compartment is postulated to occur through 1 or a combination of 3 mechanisms: paracytosis with the aid of fungal metalloproteases, transcytosis through binding between hyaluronic acid and CD44 on the endothelium, and a “Trojan horse” mechanism by hijacking host phagocytes to cross the blood-brain barrier [1].

### Virulence Factors

The *C neoformans/gattii* species complex expresses several virulence factors to enable host invasion and survival. The yeast cells are surrounded by a fungal capsule of various thickness, which is predominantly composed of the polysaccharide glucuronoxylomannan. Glucuronoxylomannan plays a pivotal role in immune modulation through inhibition of phagocytosis, phagosomal acidification, antigen presentation, T-lymphocyte proliferation and humoral response, induction of macrophage apoptosis, and induction of an immune-tolerant state [13–17]. The capsule size determines early macrophage control of infection and subsequent intracellular proliferation [18]. The production of melanin, regulated by the laccase gene, protects against intraphagocytic killing by nitrogen- and oxygen-derived radicals [19, 20]. *C neoformans* also produces a multitude of other virulence factors to aid systemic dissemination, especially CNS dissemination, including urease, hyaluronic acid, metalloprotease, and phospholipase B1 [21–24].

During in vivo infection, dramatic changes in cryptococcal cellular morphology have been observed, resulting in the formation of “titan cells,” which are 5- to 10-fold larger than typical cryptococcal yeast cells, are polypoid with a thickened cell wall and tightly compacted capsule, and form approximately 5% to 20% of the fungal cells in the infected lungs of mice [25–28]. Titan cell formation impairs phagocytosis and skews the inflammatory response to a Th2-type response [29], promoting the establishment of the initial pulmonary infection, stress adaptation, brain dissemination, and mortality [27–30].

### Host Defense

Upon inhalational exposure, cryptococcal interaction with pulmonary epithelium mainly involves adhesion mediated by glucuronoxylomannan, phospholipase B1, and the mannoprotein MP84 [31–33]. Using 2-dimensional human lung organoid derived from human embryonic stem cells, Rossi et al recently demonstrated that *C neoformans* H99 was able to invade the minilung tissue and alter the expression of surfactants [34]. In addition, pulmonary epithelia respond to cryptococcal adhesion with the production of the proinflammatory interleukin 6 (IL-6), IL-8, and CXCL1 [31, 34, 35], as well as the Th2-inducing cytokine IL-33 [36].

As the predominant resident phagocytic cells in the lung, alveolar macrophages play an essential role in the human immune response to cryptococcal invasion, including receptor-mediated phagocytosis, secretion of chemokines and cytokines, and antigen presentation, as well as serving as a reservoir for latency [37]. The ability of macrophages to contain cryptococcal invasion depends on macrophage polarization and activation status, which are influenced by the cytokine microenvironment [38]. Interferon γ (IFN-γ), tumor necrosis factor α (TNF-α), and GM-CSF signaling stimulates M1 polarization, which is essential for macrophage fungicidal activity [38, 39]. Yet, IL-4 stimulation differentially induces M2 polarization, which is associated with deficient anticryptococcal activity and disease progression [40]. Conceptually, treatments that impair M1 polarization, such as antagonists to TNF-α or JAK/STAT inhibitors (Janus kinase/signal transducer and activator of transcription) that impair IFN-γ signaling, are associated with increased risks of cryptococcosis, among a population of patients who are often already predisposed to infection due to their underlying disease or concomitant immunosuppressants.

T-cell responses after cryptococcal infection are stimulated by activated dendritic cells, which respond to fungal pathogen-associated molecular patterns such as β-glucan, chitin, and glucuronoxylomannan. Activated CD4+ T cells secrete IL-12 and IL-23 to activate the T helper 1 (Th1) cells, which in turn produce IFN-γ to “superactivate” macrophages to enhance intraphagocytic killing. However, massive accruement of pathologic cryptococcal antigen-specific Th2 cells was demonstrated in the lungs following in vivo infection, which was coordinated by lung-resident CD11b+ conventional dendritic cells and induced by cleavage of chitin by the host chitotriosidase [41].

## ASSOCIATION BETWEEN BIOLOGICS AND CRYPTOCOCCOSIS

We conducted a literature search on PubMed using combinations of an individual drug name and “cryptococcosis,” “cryptococcal,” or “cryptococcus” for publications related to cryptococcosis and biologics [42–154]. Articles containing the relevant search terms that were published from 1990 to 20 January 2024 were included for title and abstract screening. Eligible articles that contained case-level data on at least 1 individual who was receiving biologics and was diagnosed with cryptococcosis were retrieved for full-text review. References of articles containing primary data were also reviewed for additional publications that might contain patient information. Non–English-language articles, cases whose demographic and clinical details were not available, as well as data reported only in abstracts of conference proceedings or scientific meetings were excluded (Supplementary Figure 1). The list of biologics according to therapeutic targets and disease groups is summarized in Supplementary Table 1. Only biologics approved by the US Food and Drug Administration (FDA) as of 20 January 2024 were included. The definitions of proven or probable cryptococcosis followed the 2020 EORTC/MSGERC consensus definitions (European Organization for Research and Treatment of Cancer/Mycoses Study Group Education and Research Consortium) [155]. Infection was deemed “disseminated” if there was fungemia or the infection involved at least 2 noncontiguous sites.

### TNF-α Antagonists

TNF-α is a pleiotropic cytokine that is predominantly produced by cells of the monocytic lineage. It is synthesized as membrane-associated or soluble forms, and it signals through TNF receptors 1 and 2 to regulate a range of biologic activities, including inflammation, cell proliferation, host defense, and cell survival [156]. Due to the prominent role of TNF-α in the proinflammatory cascade, therapeutic targeting of the TNF pathway has been harnessed to treat various inflammatory and autoimmune conditions. Despite the revolutionary success in tackling TNF-mediated pathogenesis, the use of TNF-α antagonists has been associated with an increased risk of opportunistic infections. Due to the inhibition of the formation and maintenance of granulomas [157], TNF-α inhibition increases the risk of infection by intracellular pathogens that are normally contained by granulomatous inflammation, most notably, tuberculosis, histoplasmosis, and coccidioidomycosis [158–160].

We identified 33 published cases of proven/probable cryptococcosis associated with TNF-α antagonists: 25 cases associated with infliximab, 6 cases with adalimumab, and 1 case each with etanercept and certolizumab pegol (Table 1). There was a male preponderance (male:female ratio 2.3), and the median age was 56 years (range, 14–87). The most common indication for TNF-α antagonists was Crohn disease (14/33, 43%), followed by rheumatoid arthritis (13/33, 39%). Most patients received other immunosuppressants (27/33, 82%), including 18 (55%) with corticosteroids. The most common manifestation was pulmonary cryptococcosis (18/33, 55%), followed by disseminated cryptococcosis (7/33), cryptococcal meningitis (4/33), and skin and soft tissue infection (4/33). Except for 1 case of primary cutaneous infection associated with etanercept that was caused by *Naganishia albida* (previously *Cryptococcus albidus*) [72], all other cases were caused by *C neoformans* or speciation was not provided. The implicated TNF-α antagonist was resumed in only 2 cases: one that resulted in relapse of pulmonary cryptococcosis [55] and the aforementioned case of *N albida* primary cutaneous infection in which the patient remained well despite stopping fluconazole [72].

**Table 1.** Cases of Cryptococcosis Associated With TNF-α Antagonists

Agent: First Author	Year	Age, y	Sex	Condition	TNF-α Doses or Duration^a^	Other ISx	Manifestation	Antifungal	Outcome	Resumption of TNF-α Antagonists
Infliximab										
True [42]	2002	69	M	RA	5	Steroid, MTX	Disseminated	AmB → FLZ	Recovery	…
Hage [43]	2003	61	M	RA	3	Steroid, MTX, LFM	Pulmonary	AmB → FLZ	Recovery	…
Hrnicek [44]	2003	51	M	CD	2	Steroid, MTX	Pulmonary	AmB → FLZ	Recovery	…
Arend [45]	2004	47	F	RA	2	Steroid	Pulmonary	FLZ (5 mo)	Recovery	…
Shrestha [46]	2004	65	M	RA	3	MTX, HCQ	Pulmonary	FLZ (28 d)	Recovery	…
Muñoz [47]	2007	67	F	RA	12	Steroid, MTX	Meningitis	FLZ	Recovery	…
Kozic [48]	2008	57	M	RA	2	MTX	Disseminated	AmB	Death	…
Rehman [49]	2008	61	M	CD	2.5 y	Steroid, AZA	Pulmonary	AmB + 5FC → FLZ	Recovery	…
Arnaud [50]	2009	42	M	Sarcoidosis	2	THD, MTX, ETC (for 11 mo before INX)	Disseminated	AmB + 5FC → FLZ	Recovery	…
Kluger [51]	2009	46	M	Behçet disease	19	Steroid, MMF	Meningitis	AmB + 5FC → FLZ	Recovery	…
Osawa [52]	2010	53	M	CD	3 y	Steroid, AZA	Disseminated	AmB + 5FC → FLZ	Recovery	…
Hirai [53]	2011	39	M	CD	5	Nil	Pulmonary	Nil (surgery)	Recovery	…
Wingfield [54]	2011	70	M	RA	39	Steroid, RTX, MTX	Meningitis	AmB + 5FC → AmB → VRC (4 mo)	Recovery	…
Takazono [55]	2016	35	M	CD	8 (initial), 11 (relapse)	Steroid, 5-ASA (initial)	Pulmonary	FLZ (initial, 6 mo); FLZ → ITC + 5FC → ITC (relapse)	Relapsed after initial episode	1 mo after initial episode
Vasant [56]	2016	74	F	CD	3	Steroid	Disseminated	AmB + 5FC → VRC	Recovery	…
Yamada [57]	2016	55	M	PsO, PsA	5	Nil	Pulmonary	FLZ	Recovery	…
Asakura [58]	2017	79	M	UC	7	MTX, 5-ASA	Pulmonary	FLZ	Recovery	…
Chiriac [59]	2017	72	F	RA	20	Nil	Primary cutaneous	FLZ (3 mo)	Recovery	…
Lee [60]	2017	70	F	CD	3	Steroid, AZA	Disseminated^b^	AmB → VRC	Death	…
Nosaki [61]	2019	65	F	RA	4 y	MTX	Meningitis	AmB + 5FC → FLZ	Recovery	…
Santo [62]	2019	23	M	CD	6 mo	AZA	Pulmonary	FLZ	Recovery	…
Hussein [63]	2021	54	M	CD	5	Steroid, MTX	Pulmonary	FLZ (6 mo)	Recovery	…
Fang [64]	2023	65	M	CD	4	Nil	Pulmonary	FLZ (6 mo)	Recovery	…
		20	M	CD	22	Nil	Pulmonary	FLZ (5 mo)	Recovery	…
Sha [65]	2023	51	M	UC	4	Steroid	Pulmonary	VRC	Recovery	…
Adalimumab										
Horcajada [66]	2007	69	F	RA	26	Steroid, MTX, CQ, SSZ	Tenosynovitis	AmB + 5FC → FLZ (6 mo)	Survival (amputation)	…
Cadena [67]	2009	56	F	RA	…	MTX	Pulmonary	FLZ → AmB + 5FC → FLZ	Recovery; IRIS	…
Iwata [68]	2011	56	F	RA	10	MTX	Pulmonary	Nil (surgery)	Recovery	…
Fraison [69]	2013	54	M	AS, CD	2	Steroid, AZA	Pulmonary	AmB + 5FC → FLZ	Recovery	…
Gomes [70]	2013	87	M	RA	…	Steroid	Primary cutaneous	Surgery + FLZ (6 mo)	Recovery	…
Yeh [71]	2021	57	F	CD, SLE	3 mo	Steroid, HCQ	Pulmonary	AmB + 5FC	Recovery	…
Etanercept										
Hoang [72]	2007	14	M	PsO	8 mo	Nil	Primary cutaneous^c^	FLZ	Recovery	1 y afterward
Certolizumab pegol										
Wysocki [73]	2015	46	M	CD	…	AZA, INX (until 5 mo ago) → ADM → CZP	Disseminated	AmB + 5FC → FLZ	Recovery	…

Abbreviations: 5-ASA, 5-aminosalicylic acid; 5FC, flucytosine; ADM, adalimumab; AmB, amphotericin B; AS, ankylosing spondylitis; AZA, azathioprine; CD, Crohn disease; CQ, chloroquine; CZP, certolizumab pegol; ETC, etanercept; F, female; FLZ, fluconazole; HCQ, hydroxychloroquine; INX, infliximab; IRIS, immune reconstitution inflammatory syndrome; ISx, immunosuppressant; ITC, itraconazole; LFM, leflunomide; M, male; MMF, mycophenolate mofetil; MTX, methotrexate; PsA, psoriatic arthritis; PsO, psoriasis; RA, rheumatoid arthritis; RTX, rituximab; SLE, systemic lupus erythematosus; SSZ, sulfasalazine; THD, thalidomide; TNF-α, tumor necrosis factor α; UC, ulcerative colitis; VRC, voriconazole.

^a^Doses or duration of TNF-α antagonists before onset.

^b^Multiple infections with *Klebsiella pneumoniae* bacteremia and possible pneumocystis pneumonia.

^c^Infection by *Naganishia albida* (previously *Cryptococcus albidus*).

The risk of opportunistic infection is not equally elevated across all TNF-α antagonists. Infliximab binds to monomer and trimer forms of soluble TNF and assembles more stable complexes with soluble and transmembrane TNF, whereas etanercept binding is restricted to the trimer form, creates less stable complexes, and demonstrates lower avidity to transmembrane TNF than infliximab [161]. These differences in pharmacodynamics underlie the lower risk of opportunistic infection conferred by etanercept as compared with antibody-mediated TNF-α neutralizers such as infliximab and adalimumab, as demonstrated by data collected through the Adverse Event Reporting System of the FDA [162]. In addition, patients who develop opportunistic infections while undergoing treatment with infliximab typically manifest earlier than those taking etanercept [163]. The only study that yielded a cryptococcosis-specific risk calculation was a retrospective case-control study conducted among patients with rheumatoid arthritis who developed cryptococcosis from a single center in Taiwan over a 14-year period [164]. Though the number of cryptococcosis cases with current use of TNF-α antagonists was small, exposure to adalimumab (n = 3) was significantly associated with increased risks of cryptococcosis (adjusted odds ratio, 4.50; 95% CI, 1.03–19.66; *P* = .046) while the crude odds ratio (1.61; 95% CI, .33–7.77; *P* = .55) for etanercept (n = 2) did not reach statistical significance.

### Ibrutinib and Other Bruton Tyrosine Kinase Inhibitors

Ibrutinib is a small molecule inhibitor approved for the treatment of various lymphoid neoplasms, such as chronic lymphocytic leukemia (CLL) [165, 166], Waldenstrom macroglobulinemia [167], mantle cell lymphoma [168], and follicular lymphoma [169]. Early-onset opportunistic fungal infections have been associated with the use of ibrutinib [170], most notably cases of invasive aspergillosis with frequent involvement of the CNS [171].

Susceptibility to infection in patients treated with ibrutinib has been linked to altered B-cell receptor signaling and inhibition of IL-2–inducible kinases [172] as well as to impairments in neutrophil and monocyte functionality [173, 174]. Of note, a significant number of cases of invasive fungal infections, including cryptococcosis, in patients treated with ibrutinib occurred in heavily pretreated cases with relapsed or refractory disease [82, 175]. During experimental *C neoformans* infection with Bruton tyrosine kinase (BTK)–deficient mice, Szymczak et al found that X-linked immunodeficient mice carrying a *Btk* mutation were unable to contain *C neoformans* lung infection after intranasal inoculation and experienced disseminated disease [176]. In contrast, Messina et al found no differences in disease severity among BTK knockout mice as compared with wild type ones [177]. In addition, the administration of ibrutinib at doses replicating human exposure did not affect infection severity [177]. Collectively, these animal models and clinical data suggest that increased susceptibility to cryptococcosis in patients with BTK inhibitors (BTKis) may reflect a high net state of immunosuppression rather than sole linkage to receipt of ibrutinib [178]. Two more recent BTKis with greater specificity, acalabrutinib and zanubrutinib, are increasingly used in the treatment CLL due to better cardiovascular tolerability vs ibrutinib [179, 180]. Whether these newer BTKis are associated with the same off-target effects leading to increased susceptibility to fungal infections such as cryptococcosis is as yet unknown. Of note, however, 7 cases of cryptococcosis were reported in a pooled safety analysis of 6 studies totaling 779 patients receiving zanubrutinib [181].

We identified 28 cases of proven/probable cryptococcosis occurring in patients receiving BTKis, almost exclusively with ibrutinib (2 cases with acalabrutinib and 1 with zanubrutinib; Table 2). Only 2 cases were due to *C gattii* [88, 91]. The median age was 74 years, and 79% (22/28) were male. The main indication for receipt of BTKis was CLL (17/28, 61%), followed by mantle cell lymphoma (6/28, 21%). The median duration of treatment before onset of cryptococcosis was 4.5 months, and 18 of 28 (64.3%) cases occurred within the first 6 months of treatment. The BTKi was used as first-line therapy after diagnosis in only 29% of cases with available data (7/24) and was given with concurrent immunosuppressive treatment in 25% (6/24) of cases. The main presentations of infection were cryptococcal meningitis (10/28), pulmonary infections (9/28; including single nodule, n = 2; multiple nodules, n = 2; consolidations, n = 3; pleural empyema, n = 1), and disseminated infections (7/28). In these reports, the BTKi was inconstantly discontinued after cryptococcosis in 65% (11/17) of patients with available data, indicating a need for clearer guidelines regarding the management of these biologics after the onset of opportunistic fungal infections. With the increasing treatment options available for these lymphoid neoplasms, discontinuation of BTKis may be a reasonable approach until more data emerge.

**Table 2.** Cases of Cryptococcosis Associated With Ibrutinib and Other BTKis

Agent: First Author	Year	Age, y	Sex	Condition	BTKi as First Line	Other ISx	BTKi Before Onset, mo	Manifestation	Antifungal	Outcome	Resumption of BTKi
Ibrutinib											
Ajam [74]	2016	76	F	CLL	No	…	…	Primary cutaneous	FLZ	Recovery	…
Okamato [75]	2016	68	F	CLL	No	CHL, steroid	2	Disseminated	AmB + 5FC	Recovery	Yes
Baron [76]	2017	74	F	WM	No	CHOP, RTX, F-ara-A, CP, idelalisib,	2	Meningitis	AmB	Death	…
Kimball [77]	2017	71	M	MCL	No	RTX, bendamustine, bortezomib	4	Disseminated	AmB + 5FC	Death	…
Messina [78]	2017	88	M	LPL	No	RTX, bendamustine	1	Meningitis	AmB + 5FC	Recovery	…
		54	M	CLL	No	F-ara-A, CP, RTX	1	Disseminated	AmB + 5FC	Death	…
Sudhakaran [79]	2017	74	M	MCL	No	RTX, CHOP, bortezomib	5	Pulmonary	FLZ	Recovery	…
Sun [80]	2018	70	M	MCL	No	RTX, CHOP, bortezomib	5	Meningitis	AmB + 5FC	Recovery	…
		78	M	MCL	No	RTX, CHOP, bortezomib, bendamustine, tositumomab, Len	24	Meningitis	AmB + 5FC	Recovery	Yes
Swan [81]	2018	79	M	DLBCL	No	RTX, CHOP	2	Pulmonary	AmB + 5FC	Recovery	…
Varughese [82]	2018	70	M	CLL	Yes	Nil	5	Pulmonary	FLZ	Recovery	…
		52	M	FL	Yes	RTX	3	Pulmonary	FLZ	Recovery	…
		61	M	CLL	No	RTX, F-ara-A, CP	7	Pulmonary	FLZ	Recovery	…
Abid [83]	2019	83	M	CLL	No	F-ara-A, CP, RTX	…	Disseminated	AmB + 5FC	Recovery	…
Koehler [84]	2019	57	M	CLL	Yes	Nil	4	Pulmonary	FLZ	Recovery	…
Peri [85]	2019	82	F	CLL	Yes	RTX	8	Primary cutaneous	FLZ	Recovery	…
Stankowicz [86]	2019	66	M	CLL	No	CHL, RTX, bendamustine	5	Meningitis	AmB + 5FC	Recovery	Yes
		73	M	CLL	Yes	Steroid	2	Pulmonary	FLZ	Recovery	Yes
Brochard [87]	2020	67	M	CLL	…	…	6	Disseminated	FLZ	Death^a^	Yes
		79	M	CLL	…	…	15	Pulmonary	FLZ	Death^a^	Yes
		78	F	CLL	…	…	2	Meningitis	AmB + 5FC	Death^a^	…
Paccoud [88]	2021	88	F	CLL	No	CHL, RTX, bendamustine	8	Meningitis	AmB + 5FC	Recovery	…
Van Rooij [89]	2021	75	M	MCL	Yes	Nil	6	Disseminated	AmB + 5FC	Recovery	…
Oumayma [90]	2023	69	M	CLL	No	F-ara-A, CP, RTX	2	Meningitis	AmB + 5FC	Death	…
Sung [91]	2023	76	M	CLL	…	Nil	…	Pulmonary	FLZ	Recovery	…
Acalabrutinib											
Wilson [92]	2019	61	M	CLL	Yes	Nil	7	Meningitis	AmB + 5FC	Recovery	…
Trivedi [93]	2022	78	M	MCL	No	RTX, bendamustine	…	Meningitis	AmB + 5FC	Recovery	…
Zanubrutinib											
Patel [94]	2022	75	M	WM	No	Nil	4	Disseminated	AmB + 5FC	Death	…

Abbreviations: 5FC, flucytosine; AmB, amphotericin B; BTKi, Bruton tyrosine kinase inhibitor; CHL, chlorambucil; CHOP, cyclophosphamide, doxorubicin, vincristine, prednisolone; CLL, chronic lymphocytic leukemia; CP, cyclophosphamide; DLBCL, diffuse large B-cell lymphoma; F, female; F-ara-A, fludarabine; FL, follicular lymphoma; FLZ, fluconazole; ISx, immunosuppressant; Len, lenalidomide; LPL, lymphoplasmacytic lymphoma; M, male; MCL, mantle cell lymphoma; RTX, rituximab; WM, Waldenström macroglobulinemia.

^a^Death from unrelated causes.

### Fingolimod

Fingolimod (FTY720) is a first-in-class oral disease-modifying medication that was approved by the FDA in 2010 for the treatment of patients with relapsing forms of multiple sclerosis. It acts by interacting with sphingosine 1-phosphate receptors to prevent lymphocyte egress from lymphoid tissues, thereby reducing autoreactive lymphocyte infiltration into the CNS [182]. Fingolimod induces a rapid and reversible reduction in lymphocyte counts, which remains stable during chronic treatment at 28% and 24% of baseline values at 24 months with 0.5 and 1.25 mg, respectively [183]. Specifically, patients treated with fingolimod showed a significant reduction in circulating CD4+ T cells, and activation of T cells in the presence of fingolimod led to a subinflammatory phenotype with reduced production of IFN-γ, granzyme B, IL-17, GM-CSF, and TNF-α [184]. These perturbations in lymphocyte number and function, which predominantly impair the activation of Th1 pathways, may underlie the increased risk of cryptococcosis in patients with multiple sclerosis treated by fingolimod.

Our literature search identified 25 published cases of proven/probable cryptococcosis associated with fingolimod treatment at a median interval of 5 years (range, 1.4–12) after the initiation of therapy (Table 3). The most common presentation was cryptococcal meningitis, which occurred in 11 patients (44%), followed by disseminated infections (7/25, 28%), primary cutaneous cryptococcosis (5/25, 20%), osteomyelitis (1/25), and isolated pulmonary cryptococcosis (1/25). The median absolute lymphocyte count upon presentation was 0.3 × 10^9^/L (range, 0.09–2.39 × 10^9^/L); where available, the CD4 count ranged from 5 to 145/µL. In reported cases where the speciation of *Cryptococcus* was provided, all were caused by *C neoformans*. Fingolimod was discontinued in all cases except 1 with primary cutaneous cryptococcosis [116]. Immune reconstitution inflammatory syndrome was reported in 3 cases of cryptococcal meningitis, including a fatal case [95, 97, 104]. A search of the Novartis safety database for cases with cryptococcal meningitis between January 2006 and February 2020 identified 60 case reports, with an estimated incidence of 8 per 100 000 patient-years (95% CI, 6.0–10.0), including 13 cases with fatal outcomes [185]. Although there is currently no lymphocyte cutoff that mandates the cessation of fingolimod therapy in the prescribing information, temporary drug interruption with lymphopenia <0.2 × 10^9^/L is recommended to allow for immune reconstitution [186]. Fingolimod can be restarted when the lymphocyte count is ≥0.6 × 10^9^/L [187].

**Table 3.** Cases of Cryptococcosis Associated With Fingolimod Therapy

Manifestation: First Author	Year	Age, y	Sex	Condition	Fingolimod Duration, y	Other ISx	ALC x 10^9^/L	CD4/µL	Antifungal	Outcome
Cryptococcal meningitis										
Achtnichts [95]	2015	40s	M	RRMS	2	Nil	0.4	56	AmB + 5FC → FLZ (13 mo)	Recovery; IRIS
Grebenciucova [96]	2016	62	M	RRMS	3	Nil	0.34	…	AmB + 5FC → FLZ	Recovery
Ward [97]	2016	67	F	RRMS	3.4^a^	Nil	2.39	…	AmB → FLZ	Death; IRIS
Pham [98]	2017	61	F	RRMS	3	Nil	0.12	5	AmB + 5FC → FLZ (12 mo)	Recovery
Anene-Maidoh [99]	2018	61	F	RRMS	4.8	Nil	0.3	69	AmB + 5FC	Death
Chong [100]	2019	40	F	RRMS	2.3	Nil	0.2	…	AmB + 5FC	Recovery
Ma [101]	2020	58	M	RRMS	7	Nil	0.9	…	AmB + 5FC → FLZ	Recovery
Aoki [102]	2021	41	M	RRMS	6	Nil	0.18	…	AmB + 5FC → FLZ (1 y)	Recovery
Baghbanian [103]	2021	41	F	MS	5	Nil	0.25	…	AmB + FLZ (4 wk)	Recovery
Cuascut [104]	2021	48	F	RRMS, RA	7.6	Abatacept, HCQ	0.21	…	AmB + 5FC → FLZ	Recovery; IRIS
Nasir [105]	2023	21	F	RRMS	5	Nil	0.53	6	AmB + 5FC → FLZ	Recovery
Disseminated cryptococcosis										
Huang [106]	2015	50	M	MS	3.5	Nil	0.5	…	AmB + 5FC → FLZ	Recovery
Seto [107]	2016	63	M	MS	2	Nil	0.3	145	AmB + 5FC → FLZ	Recovery
Kaur [108]	2020	34	M	RRMS	5	Nil	…	61	AmB + 5FC → FLZ (2 y)	Recovery
Wienemann [109]	2020	49	F	RRMS	5.5	Nil	0.09	77	AmB + FLZ → AmB + 5FC → FLZ	Recovery
Kammeyer [110]	2022	61	M	RRMS	7.5	Nil	0.3	…	AmB + 5FC → FLZ	Recovery
Chey [111]	2023	56	F	MS	3.8	Nil	…	…	AmB + FLZ (12 mo)	Recovery
Zhou [112]	2023	67	M	RRMS	6	Nil	0.2	…	…	…
Primary cutaneous cryptococcosis										
Forrestel [113]	2016	62	F	MS	3	Nil	0.65	56	FLZ	Recovery
Carpenter [114]	2017	47	M	RRMS	1.4	Nil	0.3	73	FLZ (12 mo)	Recovery
Patil [115]	2020	63	M	MS	7	Nil	…	13	FLZ (6 mo)	Recovery
Dahshan [116]	2021	49	M	MS	9	Nil	0.3	…	FLZ (4 mo)	Recovery^b^
Gibson [117]	2024	33	F	RRMS	5	Nil	0.22	…	…	…
Osteomyelitis										
Carpenter [118]	2022	46	F	RRMS	12	Nil	0.3	…	AmB + 5FC → FLZ	Recovery
Pulmonary cryptococcosis										
Samudralwar [119]	2019	45	M	RRMS	3	Nil	0.68	…	FLZ	Recovery

Abbreviations: 5FC, flucytosine; ALC, absolute lymphocyte count; AmB, amphotericin B; CD4, CD4+ T-lymphocyte count; FLZ, fluconazole; HCQ, hydroxychloroquine; IRIS, immune reconstitution inflammatory syndrome; ISx, immunosuppressant; MS, multiple sclerosis; RA, rheumatoid arthritis; RRMS, relapsing remitting multiple sclerosis.

^a^Fingolimod stopped 6 to 8 weeks before onset of cryptococcosis.

^b^Fingolimod not discontinued.

### Other Biologics Associated With Cryptococcosis

In addition to the aforementioned biologics that have been shown to be associated with major risks of cryptococcosis, we identified several other biologics with ≥3 cases of treatment-associated cryptococcosis reported (Table 4). These include inhibitors of the JAK/STAT pathway, anti-CD52 antagonists, anti-CD20 antagonists, and IL-6 inhibitors. The JAK/STAT signaling pathway functions downstream of >50 cytokines and growth factors, including key players in anticryptococcal immunity, such as IFN-γ and GM-CSF [188]. STAT1 deletion resulted in a shift from Th1 to Th2 cytokine response, pronounced lung inflammation, and defective classical macrophage activation in murine models of cryptococcosis [189]. There have been 12 cases of ruxolitinib-associated cryptococcosis; most of them (8/12, 67%) did not receive other concomitant immunosuppressants, indicating that ruxolitinib per se leads to increased susceptibility to cryptococcosis. Consistent with this, in a retrospective cohort study, baricitinib (odds ratio, 12.4; 95% CI, 6.4–24.1; *P* < .0001), not dexamethasone, was associated with the development of cryptococcosis [190].

**Table 4.** Biologics With at Least 3 Cases of Treatment-Associated Cryptococcosis Reported in the Literature

Agent: First Author	Year	Age, y	Sex	Condition	Biologic Duration Before Onset	Other ISx	Manifestation	Antifungal	Outcome	Resumption of Biologic
JAK/STAT inhibitors										
• Ruxolitinib										
Wysham [120]	2013	66	M	PV, MF	18 mo	Steroid	Pulmonary	FLZ (5 mo)	Recovery	5 mo later
Chen [121]	2016	69	F	MF	46 mo	Nil	Meningitis	AmB + FLZ	Recovery	…
Hirano [122]	2017	79	M	MF	6 mo	Nil	Pulmonary	FLZ → VRC	Recovery	…
Dioverti [123]	2018	70	M	MF, cHL, HLH	12 wk	Nil	Disseminated	…	Death	…
Liu [124]	2018	71	M	CMML	3 cycles	Azacitidine, ara-C, HU	Disseminated	MIC + FLZ	Death	…
Prakash [125]	2019	51	M	PV	18 mo	Nil	Meningitis^a^	AmB + 5FC → ISA	Recovery	…
Tsukui [126]	2020	76	M	MF	5 mo	Nil	Meningitis	AmB → FLZ	Recovery	…
Kasemchaiyanun [127]	2021	56	F	MF	10 mo	Nil	Pulmonary	AmB + 5FC → FLZ	Recovery	…
Sayabovorn [128]	2021	70	M	MF	4 y	Nil	Disseminated^b^	AmB + FLZ → FLZ	Death	Continued
Ciochetto [129]	2022	82	M	MF	4 y	Steroid	Disseminated	AmB + 5FC	Death	…
Ogai [130]	2022	71	M	MF	30 mo	Nil	Disseminated^c^	Nil	Death	…
Kobe [131]	2023	77	F	NSCLC, MF	2 y	Erlotinib, ramucirumab	Pulmonary	FLZ	Recovery	…
• Tofacitinib										
Kremer [132]	2013	68	F	RA	247 d	SSZ	Pulmonary	AmB → FLZ	Recovery	…
Seminario-Vidal [133]	2015	65	M	PsO, PsA	6 mo	Steroid	Pulmonary	FLZ (6 mo)	Recovery	…
Li [134]	2024	64	F	RA	2 mo	Steroid	Disseminated	ITC, FLZ, VRC	Recovery	…
Anti-CD52 antagonist										
• Alemtuzumab										
Dilhuydy [135]	2007	44	M	CLL	6 wk	F-ara-A	Disseminated	AmB + 5FC	Death	…
Ingram [136]	2007	55	M	T-PLL	26 doses	F-ara-A, CP	Disseminated	AmB	Recovery, IRIS at 10 mo	…
Bassetti [137]	2009	70	M	CLL	6 wk	F-ara-A, CP, RTX, cyclosporin, THD	Disseminated	AmB	Death	…
Henn [138]	2014	42	M	CLL	4 doses	Steroid, F-ara-A, AC, CP, RTX	Disseminated	AmB + 5FC	Death	…
Martin-Blondel [139]	2014	60	M	CLL	…	Steroid, F-ara-A, CP, RTX	Disseminated	AmB + 5FC	Recovery	…
Cruz [140]	2019	57	F	AITL	…	Steroid, RTX, CP, AC, cyclosporin, Len	Meningitis	AmB + 5FC	Recovery	…
Anti-CD20 antagonist										
• Rituximab										
Ahmed [141]	2009	75	F	CLL	2 cycles	Steroid, CP	Meningitis	AmB + 5FC → FLZ	Recovery	…
Hirai [142]	2011	65	F	DLBCL	3 cycles	CHOP	Disseminated	FLZ → AmB + 5FC → FLZ	Recovery	52 mo later
Wingfield [54]	2011	70	M	RA	2 doses	Steroid, INX, MTX	Meningitis	AmB + 5FC → AmB → VRC (4 mo)	Recovery	…
Hamerschlak [143]	2012	62	M	DLBCL	3 cycles	CHOP	Pulmonary	FLZ	Recovery	Yes
Marchand [144]	2013	69	M	CLL	2 cycles	F-ara-A, CP	Disseminated	AmB + 5FC → FLZ	Death due to disease progression	6 mo later
AlMutawa [145]	2016	72	M	CLL, ITP	3 cycles	Steroid, F-ara-A, CP, vincristine	Disseminated	AmB + 5FC + FLZ → FLZ	Recovery	…
Patel [146]	2016	68	M	CLL	18 mo	Nil	Oral	ITC	Death due to disease progression	…
Reis [147]	2016	17	F	SLE	1 dose	Steroid, MMF	Disseminated	AmB + FLZ	Recovery	50 d later
Fontana [148]	2018	16	F	SLE	2 doses	Steroid, CP, HCQ, MMF	Meningitis	AmB + 5FC → FLZ	Recovery	…
Swan [81]	2018	79	M	DLBCL	2 cycles	CHOP, ibrutinib	Pulmonary	AmB + 5FC → FLZ (12 mo)	Recovery	Yes
Zhang [149]	2021	5	M	X-ALD, post-UCBT d130	3 doses	Cyclosporin	Meningitis	AmB + 5FC → FLZ (12 mo)	Recovery	…
Edupuganti [150]	2023	40s	F	Myositis and diffuse alveolar hemorrhage	…	Steroid	Disseminated	AmB + 5FC	Death	…
Interleukin-6 inhibitor										
• Tocilizumab										
Nishioka [151]	2018	55	M	Castleman disease	5 y	Steroid, cyclosporin	Disseminated	AmB → FLZ	Recovered	15 mo later
Khatib [152]	2021	60	M	COVID-19	3 doses	Steroid	Disseminated	AmB + 5FC	Death	…
Thota [153]	2022	76	F	COVID-19	1 dose	Steroid	Disseminated	AmB + 5FC → FLZ	Unresponsive	…
Tran [154]	2023	48	M	RA, PMR	…	Steroid, MTX	Disseminated	AmB + 5FC → FLZ	Death due to CVD	…

Abbreviations: 5FC, flucytosine; AC, anthracycline; AITL, angioimmunoblastic T-cell lymphoma; AmB, amphotericin B; ara-C, cytarabine; cHL, classical Hodgkin lymphoma; CHOP, cyclophosphamide, doxorubicin, vincristine, prednisolone; CLL, chronic lymphocytic leukemia; CMML, chronic myelomonocytic leukemia; CP, cyclophosphamide; CVD, cardiovascular disease; DLBCL, diffuse large B-cell lymphoma; F-ara-A, fludarabine; FLZ, fluconazole; HCQ, hydroxychloroquine; HLH, hemophagocytic lymphohistiocytosis; HU, hydroxyurea; INX, infliximab; IRIS, immune reconstitution inflammatory syndrome; ISA, isavuconazole; ISx, immunosuppressant; ITC, itraconazole; ITP, immune thrombocytopenia; JAK, Janus kinase; Len, lenalidomide; MF, myelofibrosis; MIC, micafungin; MMF, mycophenolate mofetil; MTX, methotrexate; NSCLC, non–small cell lung cancer; PMR, polymyalgia rheumatica; PsA, psoriatic arthritis; PsO, psoriasis; PV, polycythemia vera; RA, rheumatoid arthritis; RTX, rituximab; SLE, systemic lupus erythematosus; SSZ, sulfasalazine; STAT, signal transducer and activator of transcription; THD, thalidomide; T-PLL, T-cell prolymphocytic leukemia; UCBT, umbilical cord blood transplant; VRC, voriconazole; X-ALD, X-linked adrenoleukodystrophy.

^a^Dual infection with disseminated histoplasmosis.

^b^Dual infection with *Mycobacterium haemophilum*.

^c^Dual infection with *Mycobacterium tuberculosis*.

The anti-CD52 agent alemtuzumab is indicated in the treatment of CLL, T-cell lymphoma, and relapsing-remitting multiple sclerosis and has been used in solid organ and hematopoietic stem cell transplantation for induction therapy and acute organ rejection [191–195]. Alemtuzumab selectively targets CD52, which is expressed on the surface of B and T lymphocytes, leading to sustained lymphocyte depletion [196]. Use of alemtuzumab has been associated with a range of opportunistic infections in patients with hematologic malignancies and solid organ transplantation [197–200]. Among 547 patients with solid organ transplantation who received alemtuzumab for induction or rejection therapy, 62 (11%) experienced at least 1 opportunistic infection at a median 84 days after treatment initiation, including 2 cases of cryptococcosis [199]. Among 357 patients with CLL or cutaneous T-cell lymphoma, 33 experienced opportunistic fungal infections, including 2 cases of cryptococcosis [200]. In our review of 6 reported cases of cryptococcosis with individual case details, all occurred in heavily pretreated patients with hematologic malignancies (including 4/6 with CLL), and 5 of 6 presented with disseminated disease. Similarly, although we identified several cases of cryptococcosis in patients being treated with rituximab (anti-CD20) and tocilizumab (anti–IL-6), almost all of them received concomitant corticosteroids and/or chemotherapeutic agents, suggesting that susceptibility to cryptococcosis in these populations more likely reflected an overall degree of immunosuppression instead of the independent effect of the biologics. Other biologics with rare cases of treatment-associated cryptococcosis are included in Supplementary Table 2.

### Role of Steroid and Other Immunosuppressants in Cryptococcosis Associated With Biologics

As previously stated, a significant percentage of patients in this review received concomitant immunosuppressants, most notably corticosteroids. Increased susceptibility to infection caused by corticosteroid use is multifactorial and is influenced by corticosteroid dose and duration, as well as the underlying disease [201]. Corticosteroid use affects innate and adaptive immune responses. Specifically, corticosteroids reduce T-cell responses, particularly Th1 responses, by promoting T-cell apoptosis, suppressing T-cell activation and proliferation, and preventing cytokine production [201]. Corticosteroid use is commonly reported among specific subgroups of individuals with cryptococcosis who are immunocompromised, particularly in patients with malignancy, solid organ transplant, and autoimmune conditions [202–205]. Among HIV-seronegative cohorts with cryptococcosis, prior corticosteroid use was reported in up to 28% to 48% of patients [206–209], although the dose and duration were often not specified. Prior high-dose corticosteroid use, defined as the equivalent of ≥20 mg/d of prednisone for ≥60 days prior to diagnosis of cryptococcosis, has been associated with a higher likelihood of dissemination (41% vs 18%, *P* = .002) among patients with pulmonary cryptococcosis [210], and corticosteroid usage was associated with a higher 30-day mortality in a recent observational study from Japan [208].

Since biologics are most likely to be initiated in patients with autoimmune conditions, neoplasms, and transplantation, other immunosuppressants and immunomodulatory agents, especially those affecting the T-cell activation and proliferation pathways, play a role in mediating the risk of cryptococcosis. For example, in our identified cases, purine analogues such as fludarabine and cytarabine were often given to patients with hematologic malignancies. In addition to corticosteroids, transplant recipients are likely receiving calcineurin inhibitors, mycophenolate mofetil, and/or mTOR inhibitors (mammalian target of rapamycin), all of which affect T-cell activation and differentiation [211–213]. Therefore, the overall risk of infection is a product of the interaction between biologics and the host, as well as between biologics and *Cryptococcus* species.

There are limitations to this study. First, cases whose demographics and clinical details were not available were excluded from the analysis. Second, there is inherent difficulty in attributing causality to the biologics, as many patients in the literature review had underlying hematologic or rheumatologic conditions that impaired the immunity and they received concomitant or recent immunosuppressants, which all contributed to the increased risk of infection. The current study did not aim to address the causality of each biologic from a mechanistic point of view. Third, the manifestations of cryptococcosis may mimic other conditions. As noted in our series, different groups of biologics appeared to be associated with specific manifestations, such as the relatively high percentage of pulmonary cryptococcosis with TNF-α antagonists and skin and soft tissue infections with fingolimod. Disseminated disease most often occurred in patients receiving concomitant immunosuppressants and those with advanced age. However, there were significant differences in the exhaustivity in the diagnostic workup, which was based on the discretion of treating physicians and limited by the systematic availability of diagnostic tools. The apparent high percentage of some non-CNS forms of cryptococcosis associated with certain biologics may be partially attributed to the heterogeneity of the diagnostic workup.

## CONCLUSION

In conclusion, biologics, especially those blocking the Th1-macrophage activation pathways, impart a substantially increased risk of cryptococcosis among patient populations who are already susceptible to opportunistic infections due to their underlying conditions or concomitant immunosuppressants. With the increasing number and variety of biologics—expanding from the treatment of autoimmune diseases and neoplasms to novel therapeutics for atopy and metabolic diseases—clinicians must be vigilant of the risks, as lack of suspicion may lead to diagnostic delays and poorer outcomes. Knowledge of the association between biologic therapies and cryptococcosis, including the underlying mechanism of immune susceptibility and clinical manifestations, will help clinicians stratify the risks of cryptococcal infection and individualize the management plans for their patients. More data are needed to guide the management of cryptococcal infection in patients receiving biologic therapy, especially regarding the continuation or resumption of biologics during and after antifungal therapy.

## Supplementary Data

Supplementary materials are available at *Open Forum Infectious Diseases* online. Consisting of data provided by the authors to benefit the reader, the posted materials are not copyedited and are the sole responsibility of the authors, so questions or comments should be addressed to the corresponding author.

## Supplementary Material

ofae316_Supplementary_Data

## Notes

***Author contributions.*** X. L.: methodology, investigation, writing–original draft. O. P.: methodology, investigation, writing–original draft. K.-H. C.: writing–review and editing. K.-Y. Y.: writing–review and editing, supervision. R. M.: investigation. F. L.: supervision. M. A. S.: writing–review and editing. F. L. v. d. V.: writing–review and editing. T. B.: writing–review and editing. O. L.: conceptualization, methodology, writing–review and editing, supervision.

***Patient consent statement.*** This study exclusively uses existing published data and thus does not require ethical approval.
