# Supplementary material for: Cryptococcosis Associated With Biologic Therapy: A Narrative Review
Source: Open Forum Infect Dis. 2024 Jun 26;11(7):ofae316. doi: 10.1093/ofid/ofae316 (PMC11212009; doi:10.1093/ofid/ofae316)
Supplement: ofae316_Supplementary_Data [file ofae316_supplementary_data.zip › Supplementary File_R1.docx]

**SUPPLEMENTARY FILE**

| **Supplementary Table 1** | List of biologics included in the literature search. |
| --- | --- |
| **Supplementary Table 2** | Biologics with infrequent case reports of cryptococcosis (<3 cases in the literature). |
| **Supplementary Figure 1** | Flow diagram of search results and articles selected. |

**Supplementary Table 1. List of biologics included in the literature search.**

| **Biologic category** | **Biologic name** | **Target** | **Year of initial FDA approval** | **FDA licensed indications** | **Disease category** |
| --- | --- | --- | --- | --- | --- |
| Growth factor inhibitor mAbs | Bevacizumab | VEGF | 2004 | Metastatic CRC, NSCLC, glioblastoma, cervical cancer, metastatic RCC, epithelial ovarian, fallopian tube or peritoneal cancer | Solid tumour |
|  | Ramucirumab | VEGFR2 | 2014 | Gastric or gastroesophageal junction adenocarcinoma, NSCLC, CRC, HCC | Solid tumour |
|  | Cetuximab | EGFR | 2004 | Head and neck SCC; K-Ras wild-type, EGFR-expressing CRC | Solid tumour |
|  | Panitumumab | EGFR | 2006 | EGFR-expressing metastatic CRC | Solid tumour |
|  | Trastuzumab | HER2/ErbB2 | 1998 | HER2-overexpressing breast cancer, metastatic gastric or gastroesophageal junction adenocarcinoma | Solid tumour |
|  | Pertuzumab | HER2/ErbB2 | 2012 | HER2-overexpressing breast cancer | Solid tumour |
| Immune checkpoint inhibitors | Pembrolizumab | PD-1 | 2014 | Melanoma, NSCLC, SCLC, head and neck SCC, cHL, PMBCL, urothelial carcinoma, MSI-H or dMMR cancer including CRC, gastric cancer, oesophageal cancer, cervical cancer, HCC, MCC, RCC, EC, tumour mutational burden-high cancer, cutaneous SCC, TNBC | Solid tumour & haematological |
|  | Nivolumab | PD-1 | 2014 | Melanoma, NSCLC, malignant pleural mesothelioma, RCC, cHL, head and neck SCC, urothelial carcinoma, MSI-H or dMMR metastatic CRC, HCC, gastric cancer, gastroesophageal junction cancer, oesophageal cancer | Solid tumour & haematological |
|  | Cemiplimab | PD-1 | 2018 | Cutaneous SCC, BCC, NSCLC | Solid tumour |
|  | Dostarlimab-gxly | PD-1 | 2021 | dMMR recurrent or advanced EC and solid tumours | Solid tumour |
|  | Retifanlimab-dlwr | PD-1 | 2023 | MCC | Solid tumour |
|  | Atezolizumab | PD-L1 | 2016 | Urothelial carcinoma, NSCLC, SCLC, HCC, melanoma | Solid tumour |
|  | Durvalumab | PD-L1 | 2017 | NSCLC, SCLC, biliary tract cancer, HCC | Solid tumour |
|  | Avelumab | PD-L1 | 2017 | MCC, urothelial carcinoma, RCC | Solid tumour |
|  | Ipilimumab | CTLA-4 | 2011 | Melanoma, RCC, MSI-H or dMMR metastatic CRC, HCC, NSCLC, malignant pleural mesothelioma | Solid tumour |
|  | Tremelimumab | CTLA-4 | 2022 | HCC, NSCLC | Solid tumour |
| Kinase inhibitors | Sorafenib | Multiple kinases | 2005 | HCC, RCC, differentiated thyroid carcinoma | Solid tumour |
|  | Regorafenib | Multiple kinases | 2012 | CRC, GIST, HCC | Solid tumour |
|  | Umbralisib | Multiple kinases | 2021 | MZL, FL | Haematological |
|  | Imatinib | Multiple TKs | 2001 | Ph+ CML, Ph+ ALL, MSD/MPD, aggressive systemic mastocytosis, HES and/or CEL, dermatofibrosarcoma protuberans, Kit +ve GIST | Solid tumour & haematological |
|  | Sunitinib | Multiple TKs | 2006 | GIST, RCC, pNET | Solid tumour |
|  | Pazopanib | Multiple TKs | 2009 | RCC, soft tissue sarcoma | Solid tumour |
|  | Vendetanib | Multiple TKs | 2011 | Medullary thyroid cancer | Solid tumour |
|  | Cabozantinib | Multiple TKs | 2012 | RCC, HCC | Solid tumour |
|  | Lenvatinib | Multiple TKs | 2015 | Differentiated thyroid cancer. RCC, HCC, EC | Solid tumour |
|  | Avapritinib | Multiple TKs | 2020 | GIST, advanced systemic mastocytosis, indolent systemic mastocytosis | Solid tumour & haematological |
|  | Nintedanib | Multiple TKs | 2014 | IPF, chronic fibrosing ILD, SSc-ILD | Autoimmune |
|  | Crizotinib | ALK | 2011 | ALK or ROS1+ NSCLC, ALK+ ALCL, ALK+ inflammatory myofibroblastic tumour | Solid tumour & haematological |
|  | Ceritinib | ALK | 2014 | ALK+ NSCLC | Solid tumour |
|  | Alectinib | ALK | 2015 | ALK+ NSCLC | Solid tumour |
|  | Brigatinib | ALK | 2017 | ALK+ NSCLC | Solid tumour |
|  | Lorlatinib | ALK | 2018 | ALK+ NSCLC | Solid tumour |
|  | Axitinib | VEGFR 1-3 | 2012 | RCC | Solid tumour |
|  | Erlotinib | EGFR | 2004 | NSCLC, pancreatic cancer | Solid tumour |
|  | Osimertinib | EGFR | 2015 | NSCLC | Solid tumour |
|  | Gefitinib | EGFR | 2015 | NSCLC | Solid tumour |
|  | Lapatinib | Multiple ErbBs | 2007 | HER2-overexpressing breast cancer | Solid tumour |
|  | Afatinib | Multiple ErbBs | 2013 | NSCLC | Solid tumour |
|  | Neratinib | Multiple ErbBs | 2017 | HER2-overexpressing breast cancer | Solid tumour |
|  | Dasatinib | BCR-ABL | 2006 | Ph+ CML, Ph+ ALL | Haematological |
|  | Nilotinib | BCR-ABL | 2007 | Ph+ CML | Haematological |
|  | Bosutinib | BCR-ABL | 2012 | Ph+ CML | Haematological |
|  | Ponatinib | BCR-ABL | 2012 | CML, Ph+ ALL | Haematological |
|  | Ibrutinib | BTK | 2013 | MCL, CLL/SLL, WM, MZL, chronic GVHD | Haematological & transplant |
|  | Acalabrutinib | BTK | 2017 | MCL, CLL/SLL | Haematological |
|  | Zanubrutinib | BTK | 2019 | MCL, WM, MZL | Haematological |
|  | Idelalisib | PI3Kδ | 2014 | CLL, FL, SLL | Haematological |
|  | Alpelisib | PI3K | 2019 | PIK3CA-related overgrowth spectrum | Others |
|  | Ruxolitinib | JAK/STAT | 2011 | MF, PV, steroid-refractory GVHD | Haematological & transplant |
|  | Tofacitinib | JAK/STAT | 2012 | RA | Autoimmune |
|  | Baricitinib | JAK/STAT | 2018 | RA | Autoimmune |
|  | Fedratinib | JAK/STAT | 2019 | MF | Haematological |
|  | Pacritinib | JAK/STAT | 2022 | MF | Haematological |
|  | Deucravacitinib | JAK/STAT | 2022 | PsO | Autoimmune |
|  | Abrocitinib | JAK/STAT | 2022 | AD | Atopy |
|  | Dabrafenib | BRAF | 2013 | Melanoma, NSCLC, anaplastic thyroid cancer, and low-grade glioma with BRAF mutations | Solid tumour |
|  | Larotrectinib | TRK | 2018 | Solid tumour with NTRK gene fusion | Solid tumour |
|  | Abemaciclib | CDK4/6 | 2017 | Breast cancer | Solid tumour |
| Lymphoid cell surface antigen inhibitors | Muromonab-CD3 | CD3 | 1986 | Acute, steroid-resistant rejection of allogeneic kidney, heart, and liver transplants | Transplant |
|  | Teplizumab-mzwv | CD3 | 2022 | Type I diabetes mellitus | Metabolic |
|  | Efalizumab | CD11a | 2003 | PsO | Autoimmune |
|  | Blinatumomab | CD19 | 2014 | CD19-positive B-cell precursor ALL | Haematological |
|  | Tafasitamab-cxix | CD19 | 2020 | DLBCL | Haematological |
|  | Inebilizumab-cdon | CD19 | 2020 | AQP4 Ab-positive NMOSD | Autoimmune |
|  | Loncastuximab tesirine-lpyl | CD19 | 2021 | B-cell lymphoma | Haematological |
|  | Rituximab | CD20 | 1997 | NHL, CLL, RA, GPA, MPA, pemphigus vulgaris | Haematological & autoimmune |
|  | Ibritumomab tiuxetan | CD20 | 2002 | NHL | Haematological |
|  | Ofatumumab | CD20 | 2009 | CLL, MS | Haematological & autoimmune |
|  | Tositumomab | CD20 | 2012 | NHL | Haematological |
|  | Obinutuzumab | CD20 | 2013 | CLL, FL | Haematological |
|  | Ocrelizumab | CD20 | 2017 | MS | Autoimmune |
|  | Ublituximab-xiiy | CD20 | 2022 | MS | Autoimmune |
|  | Epcoritamab-bysp | CD20 | 2023 | DLBCL | Haematological |
|  | Glofitamab-gxbm | CD20 | 2023 | DLBCL or LBCL arising from FL | Haematological |
|  | Inotuzumab ozogamicin | CD22 | 2017 | B-cell precursor ALL | Haematological |
|  | Moxetumomab pasudotox-tdfk | CD22 | 2018 | HCL | Haematological |
|  | Brentuximab vedotin | CD30 | 2011 | cHL, systemic ALCL or other CD30-expressing PTCL, primary cutaneous ALCL or CD30-expressing mycosis fungoides | Haematological |
|  | Daratumumab | CD38 | 2015 | MM | Haematological |
|  | Isatuximab-irfc | CD38 | 2020 | MM | Haematological |
|  | Alemtuzumab | CD52 | 2001 | B-CLL, MS | Haematological & autoimmune |
|  | Polatuzumab vedotin-piiq | CD79b | 2019 | DLBCL | Haematological |
|  | Belatacept | CD80/CD86 | 2011 | Prophylaxis of organ rejection in kidney transplantation | Transplant |
|  | Belimumab | BAFF | 2011 | SLE | Autoimmune |
| TNF-α antagonists | Infliximab | TNF-α | 1998 | CD, UC, RA, AS, PsA, PsO | Autoimmune |
|  | Etanercept | TNF-α | 1998 | RA, polyarticular JIA, PsA, AS, PsO | Autoimmune |
|  | Adalimumab | TNF-α | 2002 | RA, JIA, PsA, AS, CD, UC, PsO, HS, uveitis | Autoimmune |
|  | Certolizumab | TNF-α | 2008 | CD, RA, PsA, AS, PsO | Autoimmune |
|  | Golimumab | TNF-α | 2009 | RA, PsA, AS, JIA | Autoimmune |
| Interleukin antagonists | Anakinra | IL-1 | 2001 | RA, CAPS, DIRA | Autoimmune |
|  | Rilonacept | IL-1 | 2008 | CAPS, DIRA, recurrent pericarditis | Autoimmune |
|  | Canakinumab | IL-1 | 2009 | Periodic fever syndrome, active Still's disease, gout flares | Autoimmune |
|  | Basiliximab | IL-2 | 1998 | Prophylaxis of acute organ rejection in kidney transplantation | Transplant |
|  | Denileukin diftitox | IL-2 | 1999 | CD25-positive cutaneous T-cell lymphoma | Haematological |
|  | Daclizumab | IL-2 | 2016 | MS | Autoimmune |
|  | Dupilumab | IL-4 | 2017 | AD, asthma, chronic rhinosinusitis with nasal polyposis, eosinophilic oesophagitis, prurigo nodularis | Atopy |
|  | Mepolizumab | IL-5 | 2015 | Asthma, chronic rhinosinusitis with nasal polyps, EGPA, HES | Atopy & autoimmune |
|  | Reslizumab | IL-5 | 2016 | Asthma | Atopy |
|  | Benralizumab | IL-5 | 2017 | Asthma | Atopy |
|  | Tocilizumab | IL-6 | 2010 | RA, GCA, SSc-ILD, polyarticular JIA, CRS | Autoimmune |
|  | Siltuximab | IL-6 | 2014 | MCD | Autoimmune |
|  | Sarilumab | IL-6 | 2017 | RA | Autoimmune |
|  | Satralizumab-mwge | IL-6 | 2020 | AQP4 Ab-positive NMOSD | Autoimmune |
|  | Tralokinumab-ldrm | IL-13 | 2021 | AD | Atopy |
|  | Secukinumab | IL-17A | 2015 | PsO, PsA, AS, nr-axSpA, ERA | Autoimmune |
|  | Ixekizumab | IL-17A | 2016 | PsO, PsA, AS, nr-axSpA | Autoimmune |
|  | Brodalumab | IL-17RA | 2017 | PsO | Autoimmune |
|  | Tildrakizumab-asmn | IL-23p19 | 2018 | PsO | Autoimmune |
| Complement inhibitors | Pegcetacoplan | C3 | 2021 | PNH | Haematological |
|  | Eculizumab | C5 | 2007 | PNH, aHUS | Haematological & autoimmune |
|  | Ravulizumab-cwvz | C5 | 2018 | PNH, aHUS, generalised MG | Haematological & autoimmune |
|  | Avacopan | C5 | 2021 | ANCA-associated vasculitis | Autoimmune |
| IgE inhibitor | Omalizumab | IgE | 2003 | Asthma, nasal polyp, chronic idiopathic urticaria | Atopy |
| Proteasome inhibitors | Bortezomib | Ubiquitin-proteasome pathway | 2003 | MM, MCL | Haematological |
|  | Carfilzomib | Ubiquitin-proteasome pathway | 2012 | MM | Haematological |
|  | Ixazomib | Ubiquitin-proteasome pathway | 2015 | MM | Haematological |
| α4-integrin inhibitors | Natalizumab | α4-integrins | 2004 | MS | Autoimmune |
|  | Vedolizumab | α4-integrins | 2014 | UC, CD | Autoimmune |
| Cell surface receptor glycoprotein inhibitors | Elotuzumab | SLAMF7 | 2015 | MM | Haematological |
| Others | Fingolimod | Sphingosine 1-phosphate receptor | 2010 | MS | Autoimmune |

Abbreviations: Ab – antibody; AD – atopic dermatitis; aHUS – atypical haemolytic uraemic syndrome; ALCL – anaplastic large cell lymphoma; ALL – acute lymphoblastic leukaemia; ANCA – anti-neutrophil cytoplasmic autoantibody; AQP4 – anti-aquaporin-4; AS – ankylosing spondylitis; BCC – basal cell carcinoma; BTK – Bruton tyrosine kinase; B-CLL – B-cell chronic lymphocytic leukaemia; CAPS – cryopyrin-associated periodic syndromes; CD – Crohn’s disease; CEL – chronic eosinophilic leukaemia; cHL – classical Hodgkin lymphoma; CLL – chronic lymphocytic leukaemia; CML – chronic myeloid leukaemia; CRC – colorectal cancer; CRS – cytokine release syndrome; CTLA – cytotoxic T-lymphocyte associated protein; CDIRA – deficiency of interleukin-1 receptor antagonist; DLBCL – diffuse large B-cell lymphoma; dMMR – mismatch repair deficient; EC – endometrial carcinoma; EGFR – epidermal growth factor receptor; EGPA – eosinophilic granulomatosis with polyangiitis; ERA – enthesitis-related arthritis; FDA – The United States Food and Drug Administration; FL – follicular lymphoma; GCA – giant cell arteritis; GIST – gastrointestinal stromal tumour; GPA – granulomatosis with polyangiitis; GVHD – acute graft versus host disease; HCC – hepatocellular carcinoma; HCL – hairy cell leukaemia; HES – hypereosinophilic syndrome; HER – human epidermal growth factor receptor; HS – hidradenitis suppurativa; IL – interleukin; ILD – interstitial lung disease; IPF – idiopathic pulmonary fibrosis; JAK – Janus kinase; JIA – juvenile idiopathic arthritis; LBCL – large B-cell lymphoma; mAb – monoclonal antibody; MCC – Merkel cell carcinoma; MCD – multicentric Castleman’s disease; MCL – mantle cell lymphoma; MG – myasthenia gravis; MF – myelofibrosis; MM – multiple myeloma; MPS – microscopic polyangiitis; MS – multiple sclerosis; MSD/MPD – myelodysplastic/myeloproliferative diseases; MSI-H – microsatellite instability-high; MZL – marginal zone lymphoma; NHL – non-Hodgkin’s lymphoma; NMOSD – neuromyelitis optica spectrum disorder; nr-axSpA – non-radiographic axial spondyloarthritis; NSCLC – non-small cell lung cancer; PD – programmed death; PD-L – programmed death-ligand; PI3K – phosphoinositide 3-kinases; PMBCL – primary mediastinal large B-cell lymphoma; pNET – pancreatic neuroendocrine tumour; PNH – paroxysmal nocturnal haemoglobinuria; PsA – psoriatic arthritis; PsO – plaque psoriasis; PTCL – peripheral T cell lymphoma; PV – polycythaemia vera; RA – rheumatoid arthritis; RCC – renal cell carcinoma; SCC – squamous cell carcinoma; SCLC – small cell lung cancer; SLAMF – signalling lymphocyte activation molecule family; SLE – systemic lupus erythematosus; SLL – small lymphocytic lymphoma; SSc-ILD – systemic sclerosis-associated interstitial lung disease; STAT – signal transducer and activator of transcription; TK – tyrosine kinase; TNBC – triple-negative breast cancer; UC – ulcerative colitis; VEGF – vascular endothelial growth factor; VEGFR – vascular endothelial growth factor receptor; WM – Waldenström’s macroglobulinemia.

**Supplementary Table 2. Biologics with infrequent case reports of cryptococcosis (<3 cases in the literature).**

| Agent | Author | Year | Age (yr) | Sex | Condition | Duration of biologic before onset | Other ISx | Manifestation | Antifungal | Outcome | Resumption of biologic |
| --- | --- | --- | --- | --- | --- | --- | --- | --- | --- | --- | --- |
| ***Growth factor inhibitor mAbs*** | | | | | | | | | | | |
| **Bevacizumab** | Gonzales *et al* [1] | 2021 | 48 | M | GBM | N/A | Steroid, lomustine | Disseminated | AmB + 5FC | Recovery | - |
| **Ramucirumab** | Kobe *et al* [2] | 2023 | 77 | F | NSCLC, MF | 9 months | Ruxolitinib, erlotinib | Pulmonary | FLZ | Recovery | - |
| **Cetuximab** | Calista *et al* [3] | 2015 | 74 | M | CRC | 5 cycles | FOLFOX | Gastrointestinal^a^ | AmB | Recovery | Yes (1 week later) |
| ***Immune checkpoint inhibitors*** | | | | | | | | | | | |
| **Pembrolizumab** | Patnaik *et al* [4] | 2015 | 76 | M | Melanoma | N/A | Steroid | Disseminated | N/A | Death | - |
| ***Kinase inhibitors*** | | | | | | | | | | | |
| **Erlotinib** | Kobe *et al* [2] | 2023 | 77 | F | NSCLC, MF | 9 months | Ruxolitinib, ramucirumab | Pulmonary | FLZ | Recovery | - |
| **Idelalisib** | Hengeveld *et al* [5] | 2020 | 59 | M | FL, AIHA | N/A | Steroid | Disseminated | AmB + 5FC → FLZ | Recovery | - |
| **Baricitinib** | Li *et al* [6] | 2021 | 72 | M | RA | 4 weeks | Steroid, MTX | Pulmonary | FLZ | Recovery | - |
|  | Kim *et al* [7] | 2024 | 78 | F | COVID-19 | 2 weeks | Steroid | Meningitis | AmB + 5FC → FLZ | Death | - |
| ***Cell surface receptor inhibitors*** | | | | | | | | | | | |
| **Efalizumab** | Tuxen *et al* [8] | 2007 | 34 | F | PsO, PsA | 12 months | MTX, cyclosporin | Disseminated | AmB + 5FC → FLZ (12 months) | Recovery | - |
|  | Endo *et al* [9] | 2011 | 83 | M | PsO | N/A | Topical tacrolimus | Primary cutaneous^b^ | FLZ | Recovery | - |
| **Brentuximab** | Pereira *et al* [10] | 2020 | 48 | M | cHL | 8 months | Nil | Disseminated | AmB + 5FC → FLZ | Death due to CVD | - |
| **Daratumumab** | Sato *et al* [11] | 2019 | 62 | F | MM | 3 cycles | Steroid, bortezomib, Len | Disseminated | AmB + 5FC | Death | - |
| **Abatacept** | Cuascut *et al* [12] | 2021 | 48 | F | RA, MS | N/A | Fingolimod | Meningitis | AmB + 5FC → FLZ | Recovery, IRIS | - |
|  | Mino *et al* [13] | 2021 | 73 | F | RA | N/A | Nil | Disseminated | N/A | Recovery | - |
| ***Interleukin antagonists*** | | | | | | | | | | | |
| **Ixekizumab** | Driver *et al* [14] | 2022 | 50s | M | PsO, PsA | 8 weeks | Nil | Meningitis | AmB + 5FC → VRC | Recovery | - |
| ***Complement inhibitors*** | | | | | | | | | | | |
| **Eculizumab** | Clancy *et al* [15] | 2018 | 23 | M | aHUS | 3 doses | Steroid | Disseminated | AmB + 5FC | Death | - |
|  | Lortholary *et al* [16] | 2023 | 58 | M | aHUS | 5 doses | Nil | Disseminated | FLZ | Recovery | - |
| ***IgE inhibitor*** | | | | | | | | | | | |
| **Omalizumab** | Yoshimine *et al* [17] | 2021 | 68 | M | Asthma | 3 months | Inhaled steroid | Vocal cords, trachea, and bronchi | FLZ | Recovery | - |
| ***Proteosome inhibitors*** | | | | | | | | | | | |
| **Bortezomib** | Sato *et al* [18] | 2019 | 62 | F | MM | 3 cycles | Steroid, daratumumab, Len | Disseminated | AmB + 5FC | Death | - |
| ***α4-integrin inhibitors*** | | | | | | | | | | | |
| **Natalizumab** | Valenzuela *et al* [19] | 2014 | 49 | M | RRMS | 2 years | Nil | Meningitis | AmB + 5FC → FLZ | Recovery | - |
|  | Gundacker *et al* [20] | 2016 | 46 | M | RRMS | 2 years | Steroid | Disseminated | AmB + 5FC | Death, IRIS | - |

Abbreviations: aHUS – atypical haemolytic uraemic syndrome; AIHA – autoimmune haemolytic anaemia; Amb – amphotericin B; cHL – classical Hodgkin lymphoma; COVID-19 – coronavirus disease 2019; CRC – colorectal cancer; CVD – cardiovascular disease; CFL – follicular lymphoma; FLZ – fluconazole; FOLFOX – leucovorin, fluorouracil, oxaliplatin; GBM – glioblastoma multiforme; ISx – immunosuppressant; Len – lenalidomide; mAb – monoclonal antibody; MF – myelofibrosis; MM – multiple myeloma; MTX – methotrexate; NSCLC – non-small cell lung cancer; N/A – not available; PsA – psoriatic arthritis; PsO – psoriasis; RA – rheumatoid arthritis; RRMS – relapsing-remitting multiple sclerosis; VRC – voriconazole; 5FC – 5-flucytosine.

^a^ Infection by *Papiliotrema laurentii* (previously *Cryptococcus laurentii*).

^b^ Infection by *Naganishia albida* (previously *Cryptococcus albidus*).

**Supplementary Figure 1.** Flow diagram of search results and articles selected.


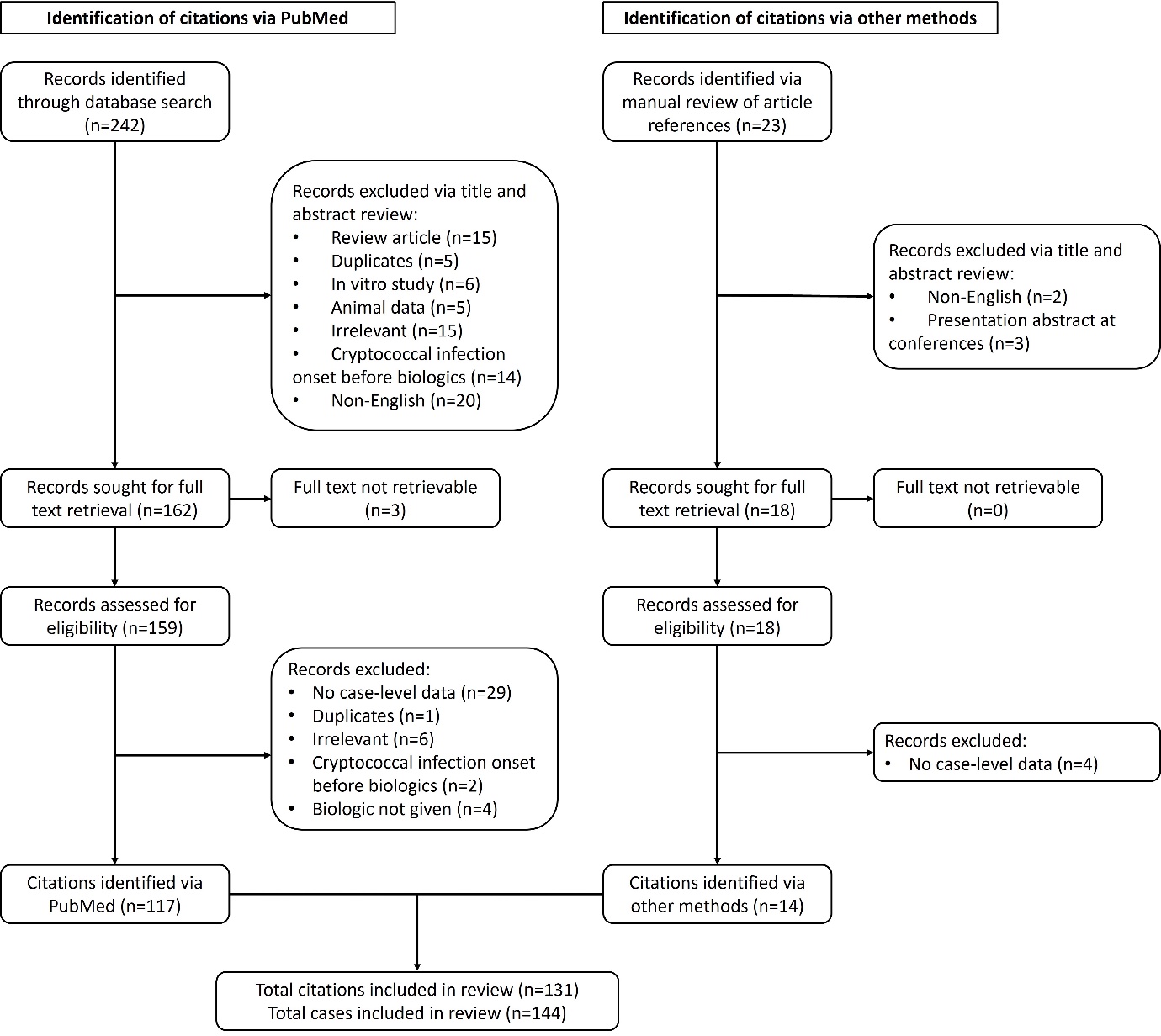


**References:**

1. Gonzales AM, Yousuf T. Disseminated cryptococcal infection in a patient with glioblastoma multiforme on treatment with lomustine and bevacizumab. Proc (Bayl Univ Med Cent) **2021**; 34(3): 376-7.

2. Kobe H, Yokoe S, Ishida T. Incidental diagnosis of pulmonary cryptococcosis by rebiopsy for epidermal growth factor receptor T790M mutation: a case report. Thorac Cancer **2023**; 14(2): 210-3.

3. Calista F, Tomei F, Assalone P, et al. *Cryptococcus laurentii* diarrhea in a neoplastic patient. Case Rep Oncol Med **2015**; 2015: 216458.

4. Patnaik A, Kang SP, Rasco D, et al. Phase I study of pembrolizumab (MK-3475; anti-PD-1 monoclonal antibody) in patients with advanced solid tumors. Clin Cancer Res **2015**; 21(19): 4286-93.

5. Hengeveld PJ, de Jongh E, Westerweel PE, Levin MD. Disseminated cryptococcal disease during treatment with idelalisib and corticosteroids for follicular lymphoma. BMJ Case Rep **2020**; 13(7).

6. Li J, Yu Y, Xie J, Jiang Y, Lu L. Cryptococcal pneumonia in a patient with rheumatoid arthritis treated with baricitinib. Rheumatology (Oxford) **2021**; 61(1): e6-e7.

7. Kim H, Kim S, Ahn MY, Oh DH, Choi JP, Yang E. Case report: cryptococcal meningitis in a previously immunocompetent patient with Coronavirus Disease 2019. Am J Trop Med Hyg **2024**; 110(2): 270-3.

8. Tuxen AJ, Yong MK, Street AC, Dolianitis C. Disseminated cryptococcal infection in a patient with severe psoriasis treated with efalizumab, methotrexate and ciclosporin. Br J Dermatol **2007**; 157(5): 1067-8.

9. Endo JO, Klein SZ, Pirozzi M, Pirozzi C, Hull CM. Generalized *Cryptococcus albidus* in an immunosuppressed patient with palmopustular psoriasis. Cutis **2011**; 88(3): 129-32.

10. Cunha Pereira T, Rb-Silva R, Félix Soares R, Domingues N, Mariz J. Case report: cryptococcal meningitis in Hodgkin's lymphoma patient receiving brentuximab-vedotin therapy. F1000Res **2020**; 9: 687.

11. Sato S, Kambe E, Tamai Y. Disseminated cryptococcosis in patient with multiple myeloma treated with daratumumab, lenalidomide, and dexamethasone. Intern Med **2019**; 58(6): 843-7.

12. Cuascut FX, Alkabie S, Hutton GJ. Fingolimod-related cryptococcal meningoencephalitis and immune reconstitution inflammatory syndrome in a patient with multiple sclerosis. Mult Scler Relat Disord **2021**; 53: 103072.

13. Mino M, Yamasaki M, Nabeshima S, et al. Cryptococcal meningitis in a patient with rheumatoid arthritis treated with abatacept. J Clin Rheumatol **2021**; 27(8s): S701-s2.

14. Driver J, Zaayman M, Silfvast-Kaiser A, Menter A. Cryptococcal meningitis associated with interleukin-17 inhibitor use for psoriasis. Dermatol Ther **2022**; 35(8): e15609.

15. Clancy M, McGhan R, Gitomer J, et al. Disseminated cryptococcosis associated with administration of eculizumab. Am J Health Syst Pharm **2018**; 75(14): 1018-22.

16. Lortholary O, El-Sissy C, Leporrier J, et al. Disseminated cryptococcosis following eculizumab therapy: insight into pathogenesis. Open Forum Infect Dis **2023**; 10(4): ofad159.

17. Yoshimine K, Tobino K, Sakabe M, Ooi R. Cryptococcosis in the vocal cords, trachea, and bronchi. Intern Med **2021**; 60(18): 3003-8.

18. Sato S, Kambe E, Tamai Y. Disseminated cryptococcosis in a patient with multiple myeloma treated with daratumumab, lenalidomide, and dexamethasone. Intern Med **2019**; 58(6): 843-7.

19. Valenzuela RM, Pula JH, Garwacki D, Cotter J, Kattah JC. Cryptococcal meningitis in a multiple sclerosis patient taking natalizumab. J Neurol Sci **2014**; 340(1-2): 109-11.

20. Gundacker ND, Jordan SJ, Jones BA, Drwiega JC, Pappas PG. Acute cryptococcal immune reconstitution inflammatory syndrome in a patient on natalizumab. Open Forum Infect Dis **2016**; 3(1): ofw038.
