# Supplementary figures and images for: Cryptococcosis Associated With Biologic Therapy: A Narrative Review
Source: Open Forum Infect Dis. 2024 Jun 26;11(7):ofae316. doi: 10.1093/ofid/ofae316 (PMC11212009; doi:10.1093/ofid/ofae316)

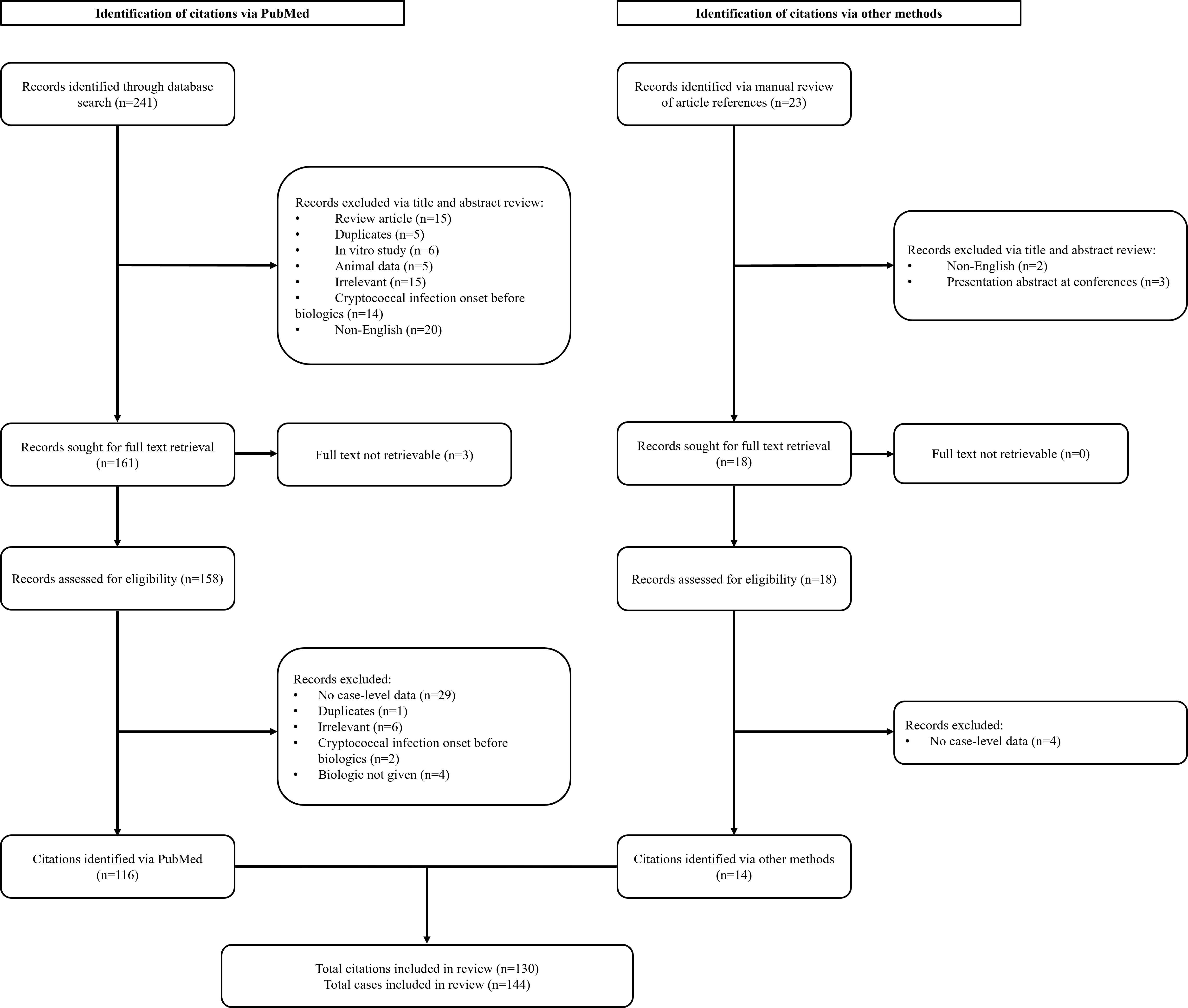

Supplement: ofae316_Supplementary_Data [file ofae316_supplementary_data.zip › OFID_R1_Supplementary-Figure-1.tif]
